# Deviant Behavior: Tick-Borne Pathogens and Inflammasome Signaling

**DOI:** 10.3390/vetsci3040027

**Published:** 2016-09-28

**Authors:** Dana K. Shaw, Erin E. McClure, Xiaowei Wang, Joao H. F. Pedra

**Affiliations:** Department of Microbiology and Immunology, University of Maryland School of Medicine, Baltimore, MD 21201, USA; erin.mcclure@umaryland.edu (E.E.M.); xiwang@som.umaryland.edu (X.W.); jpedra@som.umaryland.edu (J.H.F.P.)

**Keywords:** tick-borne diseases, tick-borne pathogens, Nod-like receptors (NLR)

## Abstract

In the face of an assault, host cells mount an immediate response orchestrated by innate immunity. Two of the best described innate immune signaling networks are the Toll- and the Nod-like receptor pathways. Extensive work has been done characterizing both signaling cascades with several recent advances on the forefront of inflammasome biology. In this review, we will discuss how more commonly-studied pathogens differ from tick-transmitted microbes in the context of Nod-like receptor signaling and inflammasome formation. Because pathogens transmitted by ticks have unique characteristics, we offer the opinion that these microbes can be used to uncover novel principles of Nod-like receptor biology.

## 1. Introduction

Innate immunity is an important first responder to infectious assaults and has a key role in facilitating the development of an adaptive immune response. The innate immune system is able to distinguish self from non-self by surveying the host milieu for pathogen-associated molecular patterns (PAMPs) or danger-associated molecular patterns (DAMPs). The different categories of innate immune signaling are grouped according to their pattern recognition receptors, which include (1) Toll-like receptors (TLR); (2) Nod-like receptors (NLR); (3) absent in myeloma (AIM2); (4) C-type lectin receptors; (5) retinoid acid-inducible gene I-like receptors (RIG I-like) and (6) cyclic GMP-AMP synthase (cGAS)/STING (stimulator of interferon genes) [1]. Two of the best studied pathways are TLR and NLR signaling, which localize and respond to stimuli either at the plasma membrane surface or intracellularly, respectively [1,2].

The field of NLR biology is rapidly advancing [3,4,5,6,7,8]. NLR proteins are cytosolic pathogen recognition receptors (PRRs) that typically contain a protein-protein interaction domain located at the N-terminus (CARD (caspase-activation and recruitment domain), BIR (baculovirus inhibitor of apoptosis protein repeat) or PYD (pyrin domain)), a central NACHT domain or Nod (nucleotide-binding oligomerization domain) and carboxy-terminal leucine-rich repeats [2]. Nod 1/2 receptors were the first characterized members of the NLR superfamily and are both activated by different forms of peptidoglycan [2,9,10]. Nod1 recognizes γ-D-glutamyl-meso-diaminopimelic acid (iE-DAP) peptidoglycan, which is typically found in the cell wall of Gram-negative bacteria, although there are a few exceptions [11,12,13]. Nod2 recognizes and binds to muramyl dipeptide (MDP), which is found in the cell wall of both Gram-negative and -positive bacteria [10,14]. More recently, both Nod1 and Nod2 were associated with potentiating an inflammatory response after endoplasmic reticulum (ER) stress was induced by the intracellular pathogen, *Brucella abortus* [15]. The adapter kinase, RIPK2, transduces the signal for both Nod1 and Nod2, which culminates in a proinflammatory immune response mediated by the transcription factors, NF-κB (nuclear factor κ B) or AP1 (activator protein 1) [2,9,16,17].

Other members of the NLR superfamily can oligomerize into a large, multi-protein scaffolding complex termed the “inflammasome”. Generally speaking, a receptor, such as an NLR, will complex with an adaptor protein and will then oligomerize. This culminates in the activation of caspase-1, which cleaves pro-IL-1β and pro-IL-18 into their mature forms. The best-studied inflammasomes are NLRP3, NLRC4, AIM2 and the noncanonical inflammasome perpetuated by caspase-11. NLRP3 inflammasomes require an NF-κB-dependent priming step, propagated by either TLR or Nod ligands, to initiate transcription of *nlrp3* [18]. The NLRP3 inflammasome contains an N-terminal PYD domain, typically requires the adapter protein ASC (apoptosis speck-like protein containing a caspase activation and recruitment domain) and is activated by a variety of stimuli, including viral infections [19], fungal products [20], bacterial RNA and DNA [21,22,23], numerous secreted bacterial products [18,24,25], crystalline or particulate matter [26,27,28], serum amyloid A and endogenous danger signals, such as ATP [24,29]. A variety of critical upstream signals have been implicated in NLRP3 activation, such as potassium efflux, fluctuations in cell volume, calcium signaling, lysosomal damage and the production of reactive oxygen species [25,28,30,31,32,33,34,35,36,37,38,39,40].

The NLRC4 inflammasome does not associate with ASC and instead complexes with NAIP molecules (NLR family, apoptosis inhibitor proteins) [3,18,41]. Upon stimulation, NAIP molecules and NLRC4 assemble into a high molecular weight, ring-shaped oligomer. Pro-caspase-1 is activated through interactions with the C-terminal CARD domain of NLRC4, which then cleaves pro-IL-1β and pro-IL-18 and induces an inflammatory form of cell death, termed pyroptosis [4,42,43]. Although there is only one NAIP encoded in the human genome, there are seven currently-known mouse NAIPs (NAIP1-7), which likely arose from gene duplication events [3,18,41]. Known stimuli that activate the NLRC4 inflammasome include flagellin (NAIP5/6) and components of the bacterial type 3 secretion systems (T3SS), such as the needle protein (NAIP1) and inner rod proteins (NAIP2). Ligands for the other NAIPs (NAIP3, 4 and 7) and their role in inflammasome activation are currently unknown [3,18,24,41].

Although not a NLR protein, the AIM2 inflammatory complex is one of the better understood inflammasomes, which recognizes cytosolic, double-stranded DNA [24,44]. AIM2 molecules have a positively-charged C-terminal HIN-200 domain, which complexes with negatively-charged double-stranded DNA, and an N-terminal PYD domain, which recruits the adapter molecule, ASC, through PYD-PYD interactions. The CARD-domain of ASC then activates procaspase-1 to induce cleavage of pro-IL-1β and pro-IL-18 [24,41]. The AIM2 inflammasome is negatively regulated by the p202 protein, which has two HIN200 domains, but which lacks a PYD domain [45,46].

Another more recently-identified pathway is the noncanonical inflammasome, which senses lipopolysaccharide (LPS) independent of TLRs. Cytosolic LPS binds directly to caspase-11 (mice) or caspase-4/5 (human), causing activation of caspase-1, secretion of IL-1β and IL-18 and pyroptosis [47]. Gasdermin D was recently identified to be downstream from caspase-11 and caspase-1 activation and to be required for both secretion of IL-1β and IL-18 and induction of pyroptosis [7,8,48,49]. Gasdermin D is cleaved by proinflammatory caspases and then forms pores from within the cell, thereby causing endogenous cell lysis, but not causing harm to neighboring cells [7,8]. Gasdermin D was also able to form pores in bacterial cell membranes, which may imply a role for the direct killing of cytosolic bacteria, although the precise role of this function remains unknown [7,8].

It is clear that inflammasome formation is crucial for host cells to sense and respond to cytosolic microbes, antigens and/or endogenous danger signals. The rapidly-advancing field of inflammasome biology highlights the complexity and importance of innate immunity. Recent advances have contributed tremendously towards our understanding. However, the field is somewhat skewed, owing to the types of microbes that have classically been used in inflammasome research. We hypothesize that there are unknown aspects of inflammasome biology, which may be elucidated in the context of infection with uncommon pathogens.

Pathogens that are transmitted by ticks are fundamentally different than some other, more commonly-studied microbes with regard to physiology, induced pathology and life strategy. For example, several tick-borne bacteria do not have canonical PAMPs, such as LPS (*Borrelia* spp., *Anaplasma* spp. and *Ehrlichia* spp.) or have modified versions that are not efficiently recognized by host PRRs (*Francisella* spp.) [50,51,52,53,54]. Many also induce a milder version of disease than what is seen from pathogens commonly used in inflammasome research. For instance, *B. burgdorferi* infects mammals and can cause persistent disease symptoms, but is not considered lethal [55,56]. Instead, these bacteria aim to avoid immune recognition, but not kill the host. This is likely a necessity given their life strategy, which involves the cyclic transmission between host and arthropod vector.

Herein, we offer the opinion that inflammasome responses differ between pathogens that are more commonly studied and tick-borne microbes. For the purpose of this review, we have selected a small subset of microbes to discuss in the context of inflammasome biology: two Gram-negative bacteria (*Salmonella* spp. and *Legionella* *pneumophila*), a genus of acid-fast bacteria (*Mycobacterium* spp.), obligate intracellular bacteria that are not vectored by arthropods (*Chlamydia* spp.) and a vector-borne parasite (*Plasmodium* spp.). These will be directly compared to five well-known tick-transmitted bacteria: *Anaplasma* spp., *Ehrlichia* spp., *Borrelia* spp., *Rickettsia* spp. and *Francisella* spp. Due to space constraints, this review will focus primarily on the most well-studied inflammasomes. For further details on inflammasome signaling in response to additional pathogens, please refer to Table 1.

## 2. Pathogens that Stimulate NLR Signaling

### 2.1. Salmonella spp.

*Salmonella* spp. are Gram-negative bacteria that are transmitted to a host via a fecal-oral route [149]. Infection is initiated by host cell invasion and replication with an endosomal compartment termed the *Salmonella*-containing vacuole (SCV), although cytosolic replication within some cell types has also been reported. This bacterium has several potent stimulators of NLR-mediated immunity, including LPS, flagellin and a T3SS [70]. Inflammasome activation against *Salmonella* has been extensively studied both in vitro and in vivo in recent years. The presence of LPS primes the NLRP3 inflammasome by stimulating TLR4 and inducing NF-κB-mediated transcription of both *nlrp3* and *pro-IL-1β* [4]. Flagellin and components of the T3SS (needle and rod proteins) stimulate the formation of the NLRC4 inflammasome and caspase-1 activation through recognition by NAIP adapter proteins [18]. Although there are discrepancies in the number of NAIPs between mice and humans, both are capable of recognizing and responding to the T3SS needle proteins and flagellin [71,72,73]. Of particular interest is the recent study published by Qu et al. providing evidence for cross-talk between NLRC4 and NLRP3 inflammasomes during *S. typhimurium* infection, which were previously believed to function independently [74]. In this study, NLRP3 is recruited to the inflammasome via the NLRC4 NACHT domain. This induces a hypothesized conformational change within NLRP3, which recruits ASC and amplifies caspase-1 activation [74].

### 2.2. L. pneumophila

*L. pneumophila* is a facultative intracellular, Gram-negative bacterium that is commonly found in aquatic reservoirs and exists in the environment by infecting amoeba [150,151,152]. If these water sources become aerosolized, *L. pneumophila* can become an accidental pathogen through inhalation and subsequent infection of alveolar macrophages [153,154]. *L. pneumophila* replicates intracellularly within an endocytic compartment and manipulates host cell biology to promote survival by injecting a plethora of effectors with the Dot/Icm (defective for organelle trafficking/intracellular multiplication) type 4 secretion system (T4SS) [155].

The NAIP5/NLRC4 inflammasome is induced in response to *L. pneumophila* flagellin and T4SS effectors, triggering a robust amount of IL-1β, IL-18 and pyroptosis [71,75,76,77,78,79,80,81,82,83]. In order for the inflammasome to be initiated, the T4SS must be intact, suggesting that flagellin may be secreted from the T4SS, although this has not yet been experimentally proven [75]. The NAIP5/NLRC4 inflammasome controls *L. pneumophila* infection with a number of mechanisms including enhanced fusion of *L. pneumophila*-containing vacuole with lysosomes, as well as flagellin-dependent activation of caspase-7, which leads to lysosome-mediated degradation of *L. pneumophila* [84,85,156]. In addition to the NAIP5/NLRC4, the ASC/NLRP3 inflammasome is also activated, although the *L. pneumophila*-derived agonist is not yet known [87,88,89].

Lastly, the non-canonical caspase-11-dependent inflammasome is also activated by *L. pneumophila* independently of flagellin, but dependent on cytosolic access of the T4SS. This inflammasome requires prior MyD88 (myeloid differentiation primary response gene 88) and TRIF (TIR-domain-containing adapter-inducing interferon-β)-induced upregulation of *caspase-11*, which leads to rapid induction of pyroptosis and the release of proinflammatory cytokines IL-1α, IL-1β and IL-18 [88,89]. Once induced, NLRP3-dependent caspase-1 activation, NAIP5/NLRC4 inflammasome activation and phagolysosomal fusion with the *L. pneumophila*-containing vacuole are enhanced [88,89,156]. Caspase-11 can also be activated by *L. pneumophila* that aberrantly enters the cytosol, likely through the recognition of LPS [157,158,159]. This was shown through the use of a mutant lacking the SdhA effector, which is needed for maintaining the integrity of the *L. pneumophila*-containing vacuole [157]. As such, the physiological relevance of this experimental model to naturally occurring infectious conditions is not clear, but can be used to understand host inflammatory processes. The Δ*sdhA* mutated bacteria are rapidly degraded, releasing double-strand DNA into the cytosol, which activates the AIM2 inflammasome and releases IL-1β. Pyroptosis is also triggered by this mutant, but is mediated by caspase-11 activation [157,158,159,160].

### 2.3. Chlamydia spp.

*Chlamydia* spp. are classified as Gram-negative and are obligate intracellular, non-flagellated bacteria that replicate within a membrane-bound endocytic compartment. Depending on the species, *Chlamydia* have a narrow range of hosts they are adapted to infect [161]. The two best studied human-adapted species are *C. trachomatis* and *C. pneumoniae* that each exhibit distinct pathologies. *C. pneumoniae* invades alveolar epithelial cells and macrophages, while *C. trachomatis* predominantly infects epithelial cells [162,163]. Host cells are manipulated by *Chlamydia* effector molecules secreted into the cytosol via a T3SS [164].

Caspase-1 activation and associated proinflammatory cytokine secretion during *C. trachomatis* infection in epithelial cells have been reported to be dependent on ASC and NLRP3, which are activated in response to potassium efflux from the cytosol, lysosomal acidification and cathepsin B release resulting from lysosomal damage [69]. However, the kinetics of inflammasome activation, how this correlates with the phase of infection, whether there is an associated benefit or detriment to the microbe and if there are variations between strains are details that are less well defined. In vitro, fibroblasts deficient in *asc* and *caspase-1* are resistant to *C. trachomatis* infection [165]. Conversely, using an in vivo infection model with the mouse-adapted species *C. muridarum*, both *caspase-1*^-/-^ and wild-type mice controlled bacterial burden comparably, although *caspase-1*^-/-^ mice had less inflammatory damage in the urogenital tract, suggesting that inflammasome activation contributes to pathology [166].

Another study performed by Abdul-Sater et al. suggested that stage-specific activation of caspase-1 directly impacted the detriment or benefit to *Chlamydia* spp. [67]. In an epithelial cell infection model with *C. trachomatis*, the translocated microbial effector Chlamydial protease-like activity factor (CPAF) inhibits ASC and caspase-1 at early time points during infection, which promoted host cell survival [165]. However, if caspase-1 was pharmacologically inhibited later in infection, bacterial replication was restricted [67]. This suggests that the kinetics of inflammasome activation can directly influence the survival of infectious microbes and the resulting pathology from disease. Moreover, it appears that pathogenic microbes can target and manipulate the kinetics of inflammasome activation to facilitate survival.

A 2015 study published by Finethy et al. proposed a model where guanylate binding proteins (GBPs) promote activation of NLRP3 and the noncanonical caspase-11-dependent inflammasomes during *Chlamydia* infection [167]. This study differentially primed macrophages with either IFN-γ or LPS and saw variations in cytokine profiles during early infection (eight hours). IL-18 secretion was dependent on the presence of GBPs, regardless of which priming agent was used (IFN-γ vs. LPS). However, GBPs were only required for secretion of IL-1β under LPS priming conditions and were dispensable for IFN-γ priming [167]. These results demonstrate that GBPs influence the kinetics of inflammasome activation and alter the relative amounts of secreted IL-1β and IL-18, which may affect the resulting pathology from *Chlamydia* infection [167].

### 2.4. Mycobacterium spp.

*Mycobacterium* spp. are acid fast bacteria with a thick cell wall consisting of mycolic acid and peptidoglycan, which contributes to the hardiness of this genus [168]. While *Mycobacterium* spp. can be environmental or pathogenic bacteria, the most well-known is the tuberculosis-causing species, *M. tuberculosis*. This pathogen enters host alveolar macrophages and replicates within an endosomal compartment by manipulating host cells with secreted effectors that prevent lysosomal fusion [169,170]. The involvement of IL-1β in host defense against *M. tuberculosis* is well established as both *il-1β* or *il-1* receptor (*ifnar1*) knockout mice are more susceptible to infection [171,172,173,174]. In the lungs of infected *ifnar1*^-/-^ mice, there is a two log-fold increase in bacteria and necrotic pneumonia develops within four weeks [174]. IL-18 also has an important role, as *il-18*^-/-^ mice were more susceptible to *M. tuberculosis* infection, but not *il-18* receptor knockouts [175].

NLRP3 has previously been implicated as the sole NLR in sensing *M.* *tuberculosis* infection, triggered by potassium depletion [176,177,178,179,180,181]. Non-tuberculous mycobacteria also stimulate NLRP3 activation, although through different mechanisms (lysosomal acidification, ROS production and cathepsin B release) [178]. The lack of AIM2 inflammasome activation during *M. tuberculosis* infection was historically perplexing because the *Mycobacterium* type VII secretion system, ESX-1, translocates DNA into the host cell cytosol [176]. However, in 2012, Saiga et al. reported that *aim2* knockout mice infected with *M. tuberculosis* had decreased levels of IL-1β and IL-18 in the lungs, suggesting that AIM2 was indeed activated [182]. In 2012, Yang et al. demonstrated that a virulent strain of *M. bovis* activated the AIM2 inflammasome from a murine-derived macrophage cell line [59], and Shah et al. observed that non-tuberculous mycobacteria elicited the AIM2 inflammasome in an ESX-1 dependent manner [183]. Interestingly, when macrophages were co-infected with *M. tuberculosis and* a non-tuberculous mycobacteria (*M. smegmatis*), the AIM2 inflammasome was suppressed [183]. This evidence suggests that *Mycobacteria* do activate the AIM2 inflammasome, but that tuberculosis-causing *Mycobacteria* have a secreted factor that suppresses inflammasome activation [176,183].

Cellular immunity has historically been considered the hallmark of immune-mediated protection against *M. tuberculosis*. These studies challenge this dogma and demonstrate that cellular-mediated immunity is not sufficient for protection, as particularly demonstrated with the *il-1* receptor knockout mice that caused an increase in bacterial burden [174,184]. This may be attributable to the role IL-1β plays in promoting the differentiation of CD4 T cells into the Th17 subtype, which have a critical role in generating an anti-*M. tuberculosis* response in addition to Th1 cells [184,185,186,187].

### 2.5. Plasmodium spp.

A hallmark of malaria pathology is the cyclic fevers that coincide with rupturing red blood cells that release merozoites during the blood stage of *Plasmodium* infection and induce a cytokine storm [188]. Proinflammatory cytokines, such as IFN-γ, TNF-α, IL-12 and, importantly, IL-1β, are produced, implicating inflammasome involvement during malaria infection [189,190,191]. Indeed, several studies have reported NLRP3 inflammasome activation, although determining the agonist has been a contentious topic [125,126,128]. Hemozoin is an inorganic crystal produced by *Plasmodium* during the heme detoxification process and has been suggested to induce the inflammasome [192]. It is released from infected erythrocytes during the asexual stage of parasite reproduction (schizogony) and has been reported to activate NLRP3 and stimulate TLR9 [193,194]. Kalantari et al. linked hemozoin to both NLRP3 and AIM2 inflammasomes [128]. NLRP3 was reportedly activated by hemozoin-induced vacuolar lysis, and the subsequent cytosolic location of hemozoin, which is bound by *Plasmodium* DNA, then stimulated the AIM2 inflammasome [128]. Whether or not the NLRP3 inflammasome contributes to malaria pathology and parasitemia has also been debated. A 2009 study published that the NLRP3 inflammasome contributes to cerebral malaria, but does not influence parasitemia [126]. Other groups have reported that, while *Plasmodium* infection stimulates NLRP3, mouse mortality caused by cerebral malaria and parasitemia were not influenced by the inflammasome [195,196,197].

Nevertheless, inflammasome induction caused by *Plasmodium* infection is indisputable. Compelling evidence for inflammasome involvement during human infection was provided in a study by Ataide et al., which showed that different subsets of patient-derived monocytes infected with either *P. vivax* or *P. falciparum* had activated forms of caspase-1 and elevated levels of secreted IL-1β [127]. Moreover, this same study demonstrated that increased expression of inflammasome genes, such as *caspase-1* and *il-1β*, were found in a *P. chabaudi* AS rodent model and correlated these phenotypes to NLRP3 and NLRP12 activation [127]. Although involvement of the inflammasome did not appear to influence the development of parasitemia or mouse survival, it did cause hypersensitivity to low doses of LPS when mice were subjected to a secondary challenge [127]. This is particularly relevant given that, in clinical settings, *Plasmodium*-infected patients are highly susceptible to a lethal septic-shock-like syndrome caused by bacterial infections [198,199,200].

## 3. Tick-Transmitted Microbes

Ticks are an ancient lineage of arthropod that diverged from insects approximately 450 million years ago. These arachnids have unique features that distinguish them from other arthropods, such as an extended life span (up to 10 years is some species) and an exclusively hematophagous diet. Lifestyle and physiology are contributing factors that allow ticks to harbor and transmit multiple pathogens of human and veterinary relevance. For example, the deer tick, *Ixodes scapularis*, can transmit up to six different pathogens, including intra- and extra-cellular bacteria, viruses and protozoa (*A.* *phagocytophilum*, an *E.* *muris-*like bacterium, *B. burgdorferi*, *B. miyamotoi*, Powassan virus, *Babesia* *microti*) [201].

Microbes that are vectored by ticks tend to deviate in life strategy and general biology from other classically-studied pathogens. Tick-borne microbes oscillate between two different environments (arthropod vs. vertebrate host) and therefore must be capable of sensing and responding to a variety of stimuli, such as fluctuations in temperature, pH, nutrient availability, dissolved oxygen levels and other undefined host/tick-specific components [202,203,204,205,206,207,208,209,210,211,212,213,214,215,216,217,218,219]. Moreover, several of these microbes have unusual physiological features, such as varied forms of LPS and peptidoglycan or the lack of LPS and peptidoglycan altogether. This is likely a reflection of the long co-evolutionary relationship that tick-borne pathogens have with the tick itself [220,221,222]. Instead, many incorporate lipids, cholesterol and lipoproteins for structural support of the cell wall [51,223,224]. These characteristics are dissimilar from typical PAMPs found on other microbes; therefore, host immune surveillance mechanisms and responses also differ from classically-described principles in immunology.

It is important to note that there are multiple variables that intermix and influence pathogen transmission of tick-borne diseases. One of the best studied variables is tick saliva and the role it has in suppressing localized host immune response. There is an extensive body of work examining the effect of tick saliva on both cellular and humoral immunity and how this ultimately influences the transmission of tick-borne microbes [225,226,227]. Saliva is crucial for promoting the prolonged feeding behavior exhibited by ticks. Effects exerted on a host by tick saliva include inhibiting itch responses, preventing blood vessel constriction and coagulation, skewing cytokine production profiles, deterring immune cell migration and differentiation and blocking wound healing [222,225,226,228,229,230,231,232,233,234,235,236,237,238,239,240,241,242,243,244,245,246,247,248,249,250,251,252,253,254,255,256,257,258]. Another more recently-published study elucidated a molecular mechanism that the tick salivary protein, sialostatin L2, has for inhibiting inflammasome formation [5]. The immunosuppressive properties of tick saliva inadvertently promote pathogen transmission to a host [227,259,260,261,262,263,264,265]. This is an important aspect to studying tick-borne microbes and understanding their life strategy. However, due to the space constraints of this review, we will limit our focus to tick-transmitted pathogens in the context of NLR and inflammasome signaling. More discussion on the immunosuppressive properties of tick saliva can be found in the following reviews [225,226,227,266,267].

### 3.1. Anaplasma spp. and Ehrlichia spp.

Both *Anaplasma* spp. and *Ehrlichia* spp. (order: Rickettsiales; family: Anaplasmataceae) are obligate intracellular bacteria that reside and replicate within an endosomal compartment that does not fuse with the lysosome. Generally speaking, *Anaplasma* spp. are transmitted by *Ixodes* spp. of ticks (*scapularis*, *ricinus*, *pacificus* and *persulcatus*), and *Ehrlichia* spp. can be transmitted by *Amblyomma americanum* (*E. chaffeensis*) and *I. scapularis* (*E. muris*-like) ticks [201,267,268]. Neither of these two bacteria have PAMPs that are known to bind Nod1/2 receptors or induce inflammasome activation; nevertheless, studies have reported that both induce NLR signaling.

A 2012 study by Sukumaran et al. demonstrated that RIPK2 (receptor interacting protein-2), the adapter kinase for Nod1/2 signaling, was a key regulator of the immune response to *A. phagocytophilum* infection in mice. Following *A. phagocytophilum* infection, *ripk2* transcripts were significantly induced above background levels. Moreover, *ripk2* knockout mice were more susceptible to infection with higher bacterial burdens, less production of pro-inflammatory cytokines, IFN-γ and IL-18, and took longer to clear *A. phagocytophilum* [108]. Subsequent studies demonstrated that *A. phagocytophilum* activates the caspase-1-dependent NLRC4 inflammasome in a manner that is reliant on annexin A2 [5]. Blocking this signaling axis by either mitigating signaling components or blocking with the tick salivary protein, sialostatin L2 (SL2), resulted in decreased caspase-1 activation and ablation of secreted IL-1β and IL-18 during *A. phagocytophilum* infection. Interestingly, these studies also demonstrated an immunosuppressive role for tick salivary proteins that are beneficial for survival and immune subversion of *A. phagocytophilum* in host cells [5,109,110].

A second study examining the mechanistic interactions between *A. phagocytophilum* and the NLRC4 inflammasome was reported recently by Wang et al. [107]. This study built on the knowledge of the annexin A2-dependent NLRC4 inflammasome triggered by *A. phagocytophilum* and demonstrated that signal transduction was dependent on the prostaglandin E2 (PGE2)-EP3 receptor axis. Upon infection, *A. phagocytophilum* activates phospholipase A2, which cleaves arachidonic acid from membrane phospholipids. Arachidonic acid is then converted to PGE2 via cyclooxygenase 2 (COX2) and the membrane-associated prostaglandin E synthase-1 (mPGES-1). EP3 receptor expression is upregulated in response to *A. phagocytophilum* infection, which subsequently binds PGE2 and propagates NLRC4 inflammasome activation and secretion of IL-1β and IL-18. In agreement with previous findings, RIPK2 was determined to be a major regulator of the immune response against *A. phagocytophilum* infection, and the loss of *ripk2* caused ablation of NF-κB and NLRC4 activation [107]. Importantly, this study demonstrated a divergence in NLRC4 pathway activation from what has been previously defined with other microbes.

Two recent studies have reported that *Ehrlichia* spp. trigger NLR signaling, which contributes to the pathology observed during fatal ehrlichiosis. Chattoraj and colleagues observed that in a murine model at seven days post-infection with the lethal ehrlichiosis-inducing isolate, *Ixodes ovatus Ehrlichia*, there was significant upregulation of genes involved in Nod-like receptor signaling (*nod2*, *nf-ΚB*, *nlrp1*, *nlrp12*, *pycard* and *il-1β*), as well as Toll-like receptor 2 (*tlr2*). Interestingly, these two signaling pathways appeared to have opposing effects on the severity of disease. Mitigation of TLR2 enhanced tissue necrosis in mice and impaired bacterial clearance, whereas loss of Nod2 led to decreased pathology, faster clearance of infection and increases in IL-10 and IFN-γ [115]. A later study published by the same group demonstrated that fatal ehrlichiosis led to increased activation of caspase-1 and caspase-11 and increased secretion of IL-1β, IL-1α and IFN-I. *Caspase-1* knockout mice were highly susceptible to disease with extensive tissue injury and increased bacterial burden. *Nlrp3*^-/-^ mice showed similar liver damage and mortality rates when compared to wild-type mice, but had decreased bacterial burden. The authors ultimately found a role for IFN-I in regulating inflammasome signaling during *Ehrlichia* infection, which appears to regulate caspase-11 activation and therefore caspase-1 dependent secretion of IL-1β [116]. Mice lacking the IFN-I receptor (*ifnar1*) were resistant to fatal disease, had lower bacterial burdens, decreased pathology and prolonged survival. The authors note that mutation of *ifnar1* during *Ehrlichia* infection led to increased autophagosomal processing. Because autophagy is a known reciprocal mechanism for regulating inflammasome activation, they hypothesize that autophagy is blocked during fatal ehrlichiosis, which leads to increased bacterial burden and pathology, owing to inflammasome activation mediated by IFNAR1 and NLRP3 signaling [116].

### 3.2. Rickettsia spp.

*Rickettsia* spp. are obligate intracellular organisms under the order Rickettsiales, but are grouped into the family Rickettsiaceae. There are several types of ticks that transmit disease-causing *Rickettsia*. Rocky Mountain spotted fever *Rickettsia* are transmitted by *Dermacentor andersoni*, *D. variabilis*, *Amblyomma* spp. and *Rhipicephalus sanguineus*, whereas Mediterranean spotted fever-causing bacteria are transmitted by only *R. sanguineus*. Unlike *Ehrlichia* spp. and *Anaplasma* spp., *Rickettsia* spp. escape the endosome and replicate within the cytoplasm of host cells.

Although *Rickettsia* are aflagellated, it is reasonable to speculate that an inflammasome or Nod1/2 response would be elicited upon *Rickettsia* infection based on the following points: (1) the cytosolic location of the bacteria; (2) the presence of both peptidoglycan and LPS in the *Rickettsia* cell wall, which are known stimulants of Nod1/2 and the caspase-11/4/5-dependent inflammasome, respectively; and (3) large amounts of secreted IL-1β and IL-18 from infected macrophages (119; personal observation). Very recently, Smalley et al. reported that *R. australis* does indeed activate caspase-1 and induces the4 secretion of IL-1β and IL-18 when bone marrow-derived macrophages are infected [119]. Secretion of these pro-inflammatory cytokines was completely abrogated in cells that were deficient in *caspase-1/caspase-11*, *asc* and *nlrp3*. Moreover, in an infection model, *nlrp3*^-/-^ mice were less capable of controlling bacterial burden in some tissues (spleen) when compared to wild-type mice, indicating that the NLRP3 inflammasome has a role in recognizing *R. australis* infection and reducing pathogen burden [119].

### 3.3. Francisella spp.

Like *Rickettsia* spp., *Francisella* spp. have peptidoglycan and LPS in the cell well and replicate within the cytosol once they have escaped the endosome [269]. However, *Francisella* spp. have an abnormal form of LPS that is tetra-acetylated and is therefore poorly recognized by TLR4 [54]. Among other host inoculation routes (i.e., aerosol), *Francisella* spp. are capable of being transmitted by *A. americanum*, *D. andersoni* and *D. variabilis* ticks. Despite the cytosolic location of these bacteria, the robust induction IL-1β and IL-18 upon infection and the presence of LPS and peptidoglycan in the bacterial cell membrane, there is no evidence for NLRP3, NLRC4 or noncanonical caspase-11-dependent inflammasome activation [117,269,270]. Instead, the AIM2 inflammasome, which is activated in response to cytosolic double-stranded DNA, is believed to respond to *Francisella* spp. infection. *Aim2* deficiency caused the ablation of caspase-1 activation, IL-1β secretion and decreased cell death. *Aim2* knockout mice were highly susceptible to *F. tularensis* infection with increased mortality rates and higher bacterial burdens [118]. A study published by Meunier et al. in 2015 demonstrated that guanylate-binding proteins (GBP) 2 and 5 had a role in inducing the lysis of *F. tularensis* in the cytosol, which would expose bacterial DNA and induce the AIM2 inflammasome [117].

### 3.4. Borrelia spp.

The Lyme disease-causing bacteria, *Borrelia* spp., are the most prevalent tick-transmitted pathogens in the United States. It is an extracellular spirochete transmitted by ticks of the *Ixodes* spp. and does not have LPS, but does have peptidoglycan, albeit an uncommon form [271]. The role of inflammasome activation in response to *Borrelia* spp. infection has not been extensively studied, owing to the extracellular nature of these bacteria. However, the inflammatory symptoms resulting from infection has prompted a few studies to examine whether inflammasome induction contributes to the pathology of Lyme disease.

In 2008, Cruz et al. reported that live *B. burgdorferi* spirochetes stimulated the release of IL-1β, which was significantly higher when compared to heat-killed bacteria [111]. These results suggested that the inflammasome could be activated in response to *B. burgdorferi* infection. A study reported by Liu et al. in 2009 examined the role that caspase-1 and ASC had during *B. burgdorferi* infection. Although the absence of caspase-1 during systemic murine infection caused an increase in bacteria burden and in the prevalence of arthritis at Day 7, these phenotypes were resolved by Day 14 [112]. In agreement with the findings from Liu et al., another group (Oosting et al.) reported caspase-1 activation in response to *B. burgdorferi* infection and secretion of IL-1β [113]. In contrast, however, this study demonstrated that capase-1 had a significant role in determining the level of joint inflammation following *B. burgdorferi* infection, characterized by cellular influx and pro-inflammatory cytokine production [113]. The differences between these studies can likely be explained by variations in time post-infection and the amount of spirochetes that were used for inoculation. While the initial 2009 study by Liu et al. evaluated the contribution of systemic infection on joint inflammation with an intradermal inoculation route of 10^4^ spirochetes, the 2011 study by Oosting et al. employed an acute inflammation model that used an intra-arterial inoculation method with 10^5^ heat-killed spirochetes delivered directly into the joint followed by a four-hour time point, post-inoculation [112,113]. The same group went on to show that with the acute model of joint swelling, using intra-arterial inoculation, but with 10^7^ live spirochetes, was significantly affected by ASC and caspase-1, but not NLRP3 [114]. While it is clear that caspase-1 is activated in response to *B. burgdorferi* infection and that this regulates the secretion of IL-1β during infection, the physiological relevance during the natural course of systemic infection leading to late Lyme disease manifestations, such as Lyme arthritis, are still unclear.

## 4. Opinion

The role that inflammasomes have in defense against invading microbes while concomitantly assisting in the development of an adaptive immune response has been well established, and the mechanisms for sensing stimuli that lead to inflammasome activation are progressively being elucidated. A significant portion of our knowledge on the mechanistic underpinnings of inflammasome signaling has been defined using a small subset of microbes, which are often well-characterized and heavily studied. While significant advances have been made with these microbes, it skews the view of inflammasome biology and leaves some areas undefined. Examples of this are the NAIPs that complex with NLRC4. Although NAIPs1, 2 and 5/6 have characterized agonists, the role that NAIPs3, 4 and 7 have in inflammasome assembly and what their respective stimuli are remain unknown [4].

Tick-transmitted microbes tend to be very different both physiologically and in pathogenicity potential from other well-characterized pathogens. This is likely an adaptation resulting from the close co-evolutionary relationship these microbes share with ticks [220,221,222]. Examples of this include the lack of, or modified versions of, LPS and peptidoglycan present in many tick-borne bacteria. Moreover, some of the more commonly-studied pathogens have secretion systems that are either direct agonists themselves (the needle and rod proteins from the T3SS elicit NLRC4) or secrete effectors that are inflammasome agonists (DNA or flagellin, which activate AIM2 or NLRP3, respectively). No such components have been described for tick-borne pathogens to date. This may be attributable to the lack of experimental data examining this question in particular. Alternatively, it is possible that tick-borne pathogens have modified or a reduced number of inflammatory PAMPs. Given the immunological pressure imparted by both mammalian and arthropod environments, which recognize common PAMPs, this is a feasible possibility. The constant selective pressure may have driven the evolutionary loss of some immunogenic components.

Despite the lack of common PAMPs, inflammasome assembly is induced in response to tick-borne infections. Tick-transmitted microbes may instead elicit inflammasome activation by inducing a dysregulated state within the host cell by causing aberrant compartmentalization of molecules, proteins and/or lipids. This could be perceived as a “danger” signal. For example, *A. phagocytophilum* directly interferes with lysosomal maturation, can take up exogenous lipids from the environment and is hypothesized to directly parasitize lipids from the host cell to sustain growth; these activities may be sufficient to induce a danger response [50,272,273,274,275,276]. NLRC4 is known to respond to *A. phagocytophilum* infection, but is not known to respond to DAMPs [5], and as such, this may represent a novel mode of inflammasome activation.

Apart from the mechanism of activation, the downstream inflammasome signaling events also seem to differ between commonly-studied microbes and tick-borne pathogens. For instance, proinflammatory cytokine secretion resulting from inflammasome induction is linked to pyroptosis when infected with more commonly-studied pathogens [4]. *A. phagocytophilum* is able to induce IL-1β and IL-18 secretion, but curiously does not induce pyroptosis [5,277]. Whether this is mediated by a microbial effector molecule to suppress inflammatory cell death or if *A. phagocytophilum* activates an unknown inflammasome mechanism that uncouples proinflammatory cytokine secretion and inflammatory cell death is not yet known. This may go hand-in-hand with what we know to be true of tick-transmitted diseases, which are associated with milder versions of pathology and are not commonly lethal, in contrast to other pathogens, such as *Salmonella* spp., *M. tuberculosis* or *Plasmodium*. Another interesting possibility is that the timing of inflammasome induction may be either detrimental or beneficial to the microbe. This hypothesis came from the observation that the *Chlamydia*-secreted effector protein, CPAF, prevents pyroptosis by inhibiting ASC and caspase-1 at early time points during infection [165]. However, if caspase-1 is blocked with pharmacological inhibitors at later time points, it restricts bacterial growth [67]. The kinetics of inflammasome activation and the correlation with the stages of intracellular bacterial replication may need to be timed correctly in order for the pathogens to establish a replicative niche and subsequently exit the host cell at an appropriate time.

With the field of inflammasome research quickly progressing, it will be enlightening to expand the arsenal of pathogenic stimuli when elucidating the mechanistic details of inflammasome formation. It is clear that there is a multitude of mechanisms for sensing inflammasome-inducing PAMPs/DAMPs, and the field has, undoubtedly, only begun to scratch the surface. By using uncommon microbes to interrogate inflammasome biology, we may be able to shed light on undiscovered mechanistic details of inflammasome activation and signal propagation, which may ultimately be correlated with disease pathology and immunological resistance to tick-borne infections.

## Figures and Tables

**Table 1:** Pathogens and associated agonists that elicit inflammasome activation.

Microbe	Organism	Gram Staining OR Phylogeny	Inflammasome	Agonist	References
Bacteria	*Mycobacterium* spp.	Acid-fast	NLRP3, AIM2	ATP, ESX-1, K+ efflux, ROS, DNA, cathepsin B release, lysosomal acidification	[57,58,59,60]
	*Bacillus anthracis*	Gram-positive	NLRP1	Lethal factor, K+ efflux	[61,62,63,64,65,66]
	*Chlamydia* spp.	Gram-negative	NLRP3	K+ efflux, cathepsin B release, ROS	[67,68,69]
	*Salmonella* spp.	Gram-negative	NLRC4, NLRP3	Flagellin, rod (PrgJ) and needle proteins	[4,18,70,71,72,73,74]
	*Legionella pneumophila*	Gram-negative	NLRC4, NLRP3	Flagellin, T4SS effectors	[71,75,76,77,78,79,80,81,82,83,84,85,86,87,88,89]
	*Shigella flexneri*	Gram-negative	NLRC4, NLRP3	Flagellin, MixI toxin	[90,91,92,93]
	Enterohemorrhagic and Enteropathogenic *Escherichia coli*	Gram-negative	NLRP3	T3SS effectors, cytoplasmic mRNA, NleA and NleE	[21,94,95,96,97,98]
	*Pseudomonas aeruginosa*	Gram-negative	NLRC4	Flagellin, mitochondrial DNA	[99,100,101,102,103]
	*Listeria monocytogenes*	Gram-positive	NLRC4, NLRP3, AIM2	Flagellin, DNA, listeriolysin O	[104,105,106]
	*Anaplasma phagocytophilum*	Gram-negative	NLRC4	Unknown	[5,107,108,109,110]
	*Borrelia burgdorferi*	Gram-negative	Unknown. ASC and caspase-1 dependent	Unknown	[111,112,113,114]
	*Ehrlichia* spp.	Gram-negative	NLRP3, caspase-11	Unknown	[115,116]
	*Francisella* spp.	Gram-negative	AIM2	dsDNA	[117,118]
	*Rickettsia* spp.	Gram-negative	NLRP3	Unknown	[119]
Parasites	*Leishmania* spp.	Kinetoplastid; vector-borne	NLRP3	K+ efflux, cathepsin B, Syk-mediated ROS production	[120,121,122]
	*Trypanosoma cruzi*	Kinetoplastid; vector-borne	NLRP3	Lysosomal damage, ROS, K+ efflux	[123,124]
	*Plasmodium* spp.	Apicomplexan; vector-borne	NLRP3, AIM2, NLRP12	Hemozoin, K+ efflux, free heme, ROS production, DNA	[125,126,127,128,129]
	*Schistosoma mansoni*	Helminth	NLRP3	ROS production, K+ efflux	[130,131]
Viruses	Hepatitis B virus (HBV)	Hepadnaviridae; dsDNA-RT	AIM2	viral dsDNA	[132]
	Hepatitis C virus (HCV)	Flavivirus; (+) RNA genome	NLRP3	K+ efflux, ROS	[133,134,135]
	Vaccinia	Orthopoxvirus; dsDNA genome	AIM2	viral dsDNA	[136]
	Respiratory syncytial virus (RSV)	Pneumovirus; (−) RNA genome	NLRP3	ROS, K+ efflux	[137]
	Rhinovirus	Enterovirus; (+) RNA genome	NLRP3, NLRC5	Ion channel protein 2B	[138]
	Dengue virus (DENV)	Flavivirus; vector-borne	NLRP3	ROS	[139,140]
	Chikungunya virus (CHIKV)	Alphavirus; vector-borne	AIM2, NLRP3	Unknown	[141,142]
	Human immunodeficiency virus 1 (HIV-1)	Lentivirus; (+) RNA genome	NLRP3	Cathepsin B, ROS, K+ efflux	[135,143,144,145]
	Influenza A (IAV)	Influenza virus A; (−) RNA genome	NLRP3	ROS, lysosomal maturation, K+ efflux	[22,146,147]
	Herpes simplex virus 1 (HSV-1)	Simplex virus; dsDNA	NLRP3, AIM2	dsDNA	[23,148]
